# Mediastinoscopy-Assisted Esophagectomy as an Effective Treatment for IgG4-Related Esophageal Stenosis: A Case Report

**DOI:** 10.70352/scrj.cr.25-0346

**Published:** 2025-07-29

**Authors:** Masazumi Sakaguchi, Shigeru Tsunoda, Mitsuhiro Nikaido, Yuki Teramoto, Shintaro Okumura, Midori Hara, Shoichi Kitano, Kohei Ueno, Ryuhei Aoyama, Yu Yoshida, Takehito Yamamoto, Takashi Sakamoto, Masahiro Maeda, Keiko Kasahara, Ryosuke Okamura, Nobuaki Hoshino, Yoshiro Itatani, Shigeo Hisamori, Koya Hida, Kazutaka Obama

**Affiliations:** 1 Department of Surgery, Graduate School of Medicine, Kyoto University, Kyoto, Kyoto, Japan; 2 Department of Gastroenterology, Tenri Hospital, Tenri, Nara, Japan; 3 Department of Diagnostic Pathology, Graduate School of Medicine, Kyoto University, Kyoto, Kyoto, Japan

**Keywords:** IgG-4 related disease, esophageal stenosis, mediastinoscopy-assisted esophagectomy

## Abstract

**INTRODUCTION:**

Immunoglobulin G4-related disease (IgG4-RD) rarely involves the esophagus, typically causing stenosis that presents significant diagnostic and therapeutic challenges. Due to its rarity and its mimicry of other conditions, obtaining a definitive preoperative diagnosis can be difficult. This report details a case of IgG4-RD-induced esophageal stenosis with initial diagnostic ambiguity, which was successfully managed with mediastinoscopy-assisted esophagectomy (MAE), highlighting this minimally invasive approach in a patient with comorbidities.

**CASE PRESENTATION:**

A 70-year-old male with comorbidities, including obstructive pulmonary disorder, presented with progressive dysphagia and epigastric discomfort. Endoscopy revealed a persistent mid-esophageal ulcer and a non-passable circumferential stricture; multiple biopsies were nondiagnostic for malignancy or infection. Given the refractory nature of the stenosis, MAE with gastric conduit reconstruction was performed. The postoperative course was uneventful, and the patient achieved symptom resolution without medication. Histopathological examination of the resected esophagus confirmed IgG4-RD, showing obliterative phlebitis and a dense infiltrate of IgG4-positive plasma cells (80/high-power field; IgG4/IgG ratio 80/85).

**CONCLUSIONS:**

This case underscores that IgG4-RD should be considered in the differential diagnosis of refractory esophageal stenosis, even with initially inconclusive biopsies. While serum IgG4 measurement has low sensitivity, it is still recommended. For benign esophageal stenosis of unclear etiology, particularly in patients with significant comorbidities, MAE can be a useful and potentially curative surgical option, offering symptom resolution and the possibility of a drug-free outcome.

## Abbreviations

CMV-PCR
: cytomegalovirus polymerase chain reaction
COPD
: chronic obstructive pulmonary disease
EoE
: eosinophilic esophagitis
EVG
: elastic van Gieson
GERD
: gastroesophageal reflux disease
HPF
: high-power field
IgG
: immunoglobulin G
IgG4
: immunoglobulin G4
IgG4-RD
: immunoglobulin G4-related disease
MAE
: mediastinoscopy-assisted esophagectomy

## INTRODUCTION

Immunoglobulin G4-related disease (IgG4-RD) is a systemic chronic immune-mediated condition characterized by infiltration with IgG4-positive plasma cells and is often accompanied by elevated serum IgG4 level.^1–3)^ While the pancreas, salivary glands, and bile ducts are commonly affected, esophageal involvement is rare.^2,4)^ When the esophagus is affected, the most clinically significant consequence is the development of esophageal stenosis or strictures.^4–6)^ Patients may present with progressive dysphagia and odynophagia, which can severely compromise oral intake and nutritional status.^4,5,7)^ Diagnosis relies on a combination of clinical suspicion, histopathological findings, imaging, and serologic testing.^2,3,5,6)^ Treatment typically involves glucocorticoid therapy, although fibrotic stricture may be resistant, often necessitating additional immunosuppression and endoscopic interventions.^4,6,8)^ Due to its rarity and overlapping features with other esophageal disorders, IgG4-related esophageal disease poses a diagnostic and therapeutic challenge.^5,6)^ In this report, we presented a case of esophageal stenosis secondary to IgG4-RD, highlighting the complexities in its diagnosis and management.

## CASE PRESENTATION

**Fig. 1:**
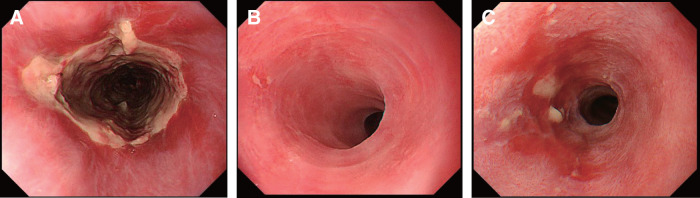
Representative endoscopic findings. (**A**) Initial presentation: Circumferential ulceration was observed. (**B**) Remission phase: No mucosal damage or stenosis was observed. (**C**) Relapse phase: Erosion and mild stenosis were noted.

**Fig. 2:**
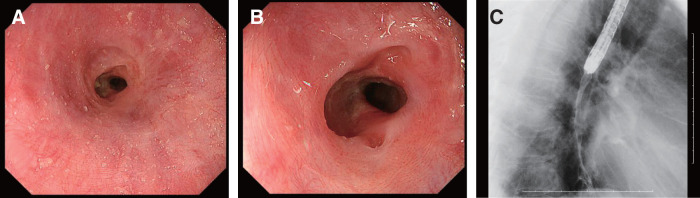
Current endoscopic findings. Stenosis and a deep ulcer were observed at the same location as previous findings. (**A**) Distant view. (**B**) Close-up view. (**C**) X-ray fluoroscopy: A severe 3-cm-long stricture was present at 35 cm from the incisors.

**Fig. 3:**
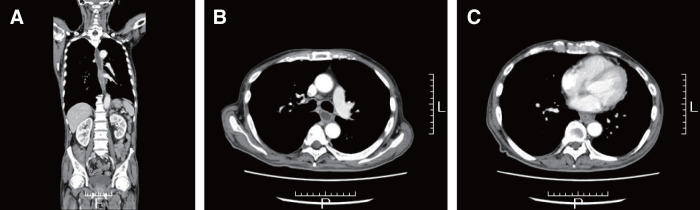
CT findings. Circumferential stenosis was observed in the same segment as the endoscopic findings. (**A**) Sagittal view. (**B**) Dilatation of the proximal esophagus was noted. (**C**) Circumferential stenosis.

A 70-year-old male with a medical history of chronic obstructive pulmonary disease, diabetes mellitus, alcoholic liver disease, and dyslipidemia initially presented with dysphagia, which progressed to epigastric discomfort. Subsequent endoscopic evaluations revealed a persistent mid-esophageal ulcer (28–35 cm from the incisors) with associated stenosis (**Fig. 1A**). Biopsy showed no malignancy, and a potassium-competitive acid blocker was introduced under the provisional diagnosis of gastroesophageal reflux disease (GERD). However, despite empirical therapy, including acid suppression and discontinuation of suspected medications, the lesion persisted, with repeated biopsies and CMV-PCR testing negative for malignancy or infection. Although transient improvement was observed over the following year (**Fig. 1B** and **1C**), dysphagia gradually worsened, and endoscopy demonstrated a non-passable, circumferential esophageal stricture with ulceration (**Fig. 2A**–**2C**). CT showed no tumors or other diseases except for mid-esophageal wall thicknning (**Fig. 3**). Given the refractory nature of the condition, surgical management was ultimately proposed.

**Fig. 4:**
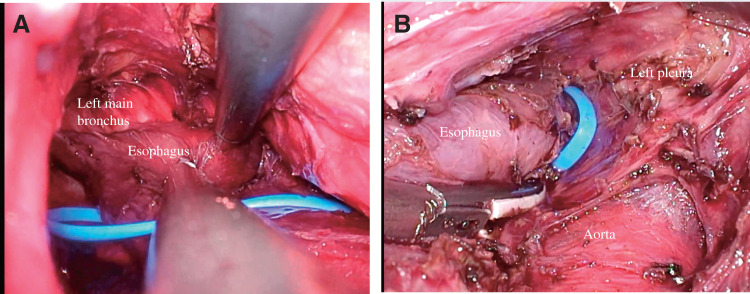
Surgical findings. Mediastinoscopy-assisted transcervical and transhiatal esophagectomy was performed. (**A**) Transcervical view: The cervical and upper esophagus were mobilized and encircled with a tape around the carina. (**B**) Transhiatal view: The lower and middle esophagus were mobilized using the previously placed tape as a guide.

Mediastinoscopy-assisted transcervical and transhiatal esophagectomy^9,10)^ with gastric conduit and cervical anastomosis was performed, considering the patient had obstructive pulmonary disorder. First, the cervical and upper esophagus were mobilized and encircled with a tape as caudally as possible via transcervical approach (**Fig. 4A**). Then, transhiatal dissection was performed, and the lower and middle esophagus were mobilized using the previously placed tape as a guide (**Fig. 4B**). Finally, the gastric conduit was pulled up via the posterior sternal route, and the esophagogastric anastomosis was performed. The postoperative course was uneventful, and the patient was discharged without complications. He remains asymptomatic without medication.

**Fig. 5:**
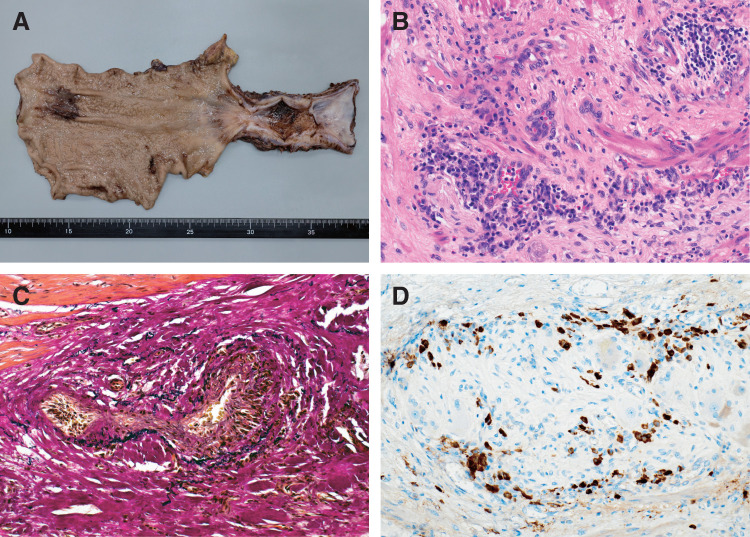
Histopathological features of the resected esophagus. (**A**) Macroscopic findings: Circumferential stenosis with ulceration is observed in the lower esophagus. (**B**) Hematoxylin and eosin staining shows dense submucosal fibrosis accompanied by a plasma cell–rich inflammatory infiltrate (objective magnification, ×20). (**C**) Elastic van Gieson staining highlights obliterative phlebitis, with concentric fibrotic occlusion of the vascular lumen (×20). (**D**) Immunohistochemistry for IgG4 reveals numerous IgG4-positive plasma cells, exceeding 50 cells per high-power field in hotspot areas (×20). IgG4, immunoglobulin G4

Histopathological examination of the resected esophageal tissue revealed mucosal ulceration accompanied by marked fibrosis extending through the full thickness of the wall (**Fig. 5A** and **5B**), although the muscularis propria was largely preserved. Elastic van Gieson (EVG) staining demonstrated fibrous obliteration of venous lumens with preserved elastic laminae, consistent with obliterative phlebitis (**Fig. 5C**). A dense, plasma cell-predominant inflammatory infiltrate with prominent perineural involvement extended into the adventitia (**Fig. 5B**). Immunohistochemical analysis showed numerous IgG4-positive plasma cells, with up to 80 cells per high-power field (HPF) observed in the most densely infiltrated (hot spot) areas (**Fig. 5D**). The IgG4/IgG-positive plasma cell ratio in these areas reached 94% (80/85). While storiform fibrosis was not evident, the presence of obliterative phlebitis along with dense IgG4-positive plasma cell infiltration fulfilling the quantitative threshold supported the diagnosis of IgG4-related disease. The lesion showed extensive transmural involvement, providing a pathological basis for the stricture’s resistance to conservative treatment.

## DISCUSSION

In this report, we described a case of esophageal stenosis due to IgG4-RD, which was challenging to diagnose and was successfully treated with mediastinoscopy-assisted transcervical and transhiatal esophagectomy, achieving a favorable outcome. IgG4-RD is an increasingly recognized systemic fibroinflammatory condition characterized by tissue infiltration of IgG4-positive plasma cells and often, though not invariably, elevated serum IgG4 levels.^1,3,5,6)^ While IgG4-RD can affect virtually any organ system,^1,3)^ esophageal involvement remains a rare manifestation,^5,6)^ posing considerable diagnostic and therapeutic challenges.

IgG4-RD of the esophagus is exceptionally rare, with only a handful of cases reported in the literature to date. A retrospective study by Obiorah et al. identified just 8 cases over a 6-year period, while Oh et al. noted only 3 previously published cases as of 2015.^5,6)^ A significant challenge in managing IgG4-RD of the esophagus is the difficulty in establishing a definitive diagnosis.^6)^ The clinical presentation can be insidious and varied, often mimicking other conditions such as malignancy or simple reflux esophagitis.^1,6)^ Patients may present with dysphagia,^5,6)^ odynophagia, or symptoms of gastroesophageal reflux.^5)^ Endoscopic findings are equally diverse, ranging from strictures, nodules, and ulcerations to, in some instances, an apparently unremarkable mucosa.^5,6)^ The hallmark histopathological features of IgG4-RD—a dense lymphoplasmacytic infiltrate, storiform fibrosis, and obliterative phlebitis, with an abundance of IgG4-positive plasma cells (>50/HPF, IgG4/IgG ratio >40%)—are essential for diagnosis.^1,5,6)^ However, these features—particularly obliterative phlebitis—may not always be evident in small or superficial biopsy specimens,^5)^ potentially leading to diagnostic delays or misinterpretations. Obtaining adequate tissue for a conclusive histological diagnosis via endoscopic biopsy can be challenging. In the present case, despite multiple biopsies, a definitive diagnosis could not be established from the collected specimens. This was due to the insufficient quantity of tissue obtained. Furthermore, serum IgG4 levels are not elevated in a substantial proportion of patients (nearly 50%),^1,5)^ diminishing its utility as a standalone diagnostic marker and underscoring the reliance on meticulous histopathological assessment. In this case, serum IgG4 was not measured preoperatively; however, when measured postoperatively, it was 45.7 mg/dL (reference range: 11–125 mg/dL) and was not elevated. Limited recognition of esophageal involvement by IgG4-RD, both clinically and histologically, may further delay appropriate management, particularly in atypical organ sites.^6)^

When initial investigations cannot reach a definitive diagnosis, a systematic approach considering various differential diagnoses is important (**Table 1**). Esophageal malignancy is a primary concern, typically presenting as an irregular mass or ulcerated stricture on endoscopy with clinical symptoms, such as pain and weight loss. Biopsies show dysplastic or malignant cells, and treatment involves surgery, chemotherapy, and radiotherapy depending on the stage.^1,6,11–13)^ Peptic strictures, secondary to chronic GERD, are next. Endoscopy often shows smooth, tapered narrowing, particularly in the distal esophagus. Histology reveals chronic inflammation and fibrosis, and treatments include proton pump inhibitors/potassium-competitive acid blockers and endoscopic dilatation.^14)^ Eosinophilic esophagitis (EoE) can cause strictures, and its endoscopic findings are concentric rings, linear furrows, or white exudates. Biopsies show significant eosinophilic infiltration (>15 eosinophils/HPF). Treatments include dietary elimination, topical corticosteroids, or biologics.^5,15)^ Finally, IgG4-RD should also be included in the differential diagnosis of benign esophageal strictures, particularly when biopsies are nondiagnostic and the lesion is refractory to standard medical therapy. Unlike malignancy or EoE, IgG4-RD may exhibit dense submucosal fibrosis and architectural changes that are difficult to appreciate in superficial samples.

**Table 1:** Esophageal stenosis

Differential diagnosis	Endoscopic findings	Pathological findings	Treatment	Reference
Esophageal malignancy	Irregular mass, ulceration, nodularity, luminal narrowing, friable mucosa	Dysplasia, adenocarcinoma, squamous cell carcinoma cells	Surgery, chemotherapy, radiotherapy, endoscopic resection/ablation (stage-dependent)	^1,6,11–13)^
Peptic stricture (GERD-related)	Smooth, tapered narrowing, often distal esophagus; associated esophagitis, hiatus hernia	Chronic inflammation, fibrosis, ulceration; rule out Barrett's esophagus, dysplasia	High-dose PPIs, endoscopic dilation, anti-reflux surgery (refractory cases)	^14)^
EoE	Fixed or dynamic rings (trachealization), linear furrows, white exudates/plaques, edema, stricture	Squamous epithelium infiltrated by eosinophils (≥15 eosinophils/HPF), eosinophilic microabscesses, basal cell hyperplasia	Dietary elimination (e.g., 6-food elimination diet), topical swallowed corticosteroids, PPIs, endoscopic dilation	^5,15)^
IgG4-related esophageal disease	Stricture, submucosal tumor-like lesion, diffuse wall thickening, nodularity, friable mucosa	Dense lymphoplasmacytic infiltrate, storiform fibrosis, obliterative phlebitis, increased IgG4+ plasma cells (>50/HPF, IgG4/IgG ratio >40%)	Systemic corticosteroids (e.g., prednisone), immunosuppressants (e.g., azathioprine, mycophenolate, rituximab)	^1,3,5,6)^

GERD, gastroesophageal reflux disease; PPI, proton pump inhibitors; EoE, eosinophilic esophagitis; HPF, high-power field; IgG4, immunoglobulin G4

Once diagnosed, IgG4-RD typically demonstrates a gratifying response to systemic corticosteroids, which remain the cornerstone of induction therapy.^1,3,5,6)^ Many patients experience significant clinical and radiological improvement with steroid treatment.^5,6)^ However, relapse upon steroid tapering or cessation is common,^5)^ necessitating long-term maintenance therapy with low-dose corticosteroids or the introduction of steroid-sparing immunosuppressive agents such as mycophenolate mofetil, azathioprine, or rituximab.^1,3,5)^ These agents have shown efficacy in maintaining remission and reducing the cumulative burden of steroid-related side effects.^1,5)^

While medical management is the established first-line approach for IgG4-RD,^1,3,5)^ the role of surgery in specific presentations warrants consideration. Historically, surgical intervention, often esophagectomy, for IgG4-related esophageal disease was typically reserved for cases with severe, refractory strictures or when malignancy could not be confidently excluded.^5,6)^ Indeed, early diagnosis and appropriate medical therapy can often obviate the need for major surgical procedures and prevent unwarranted esophagectomies.^5)^

However, particularly in instances where IgG4-RD is demonstrably localized to the esophagus, as in the present case, surgical resection offers a distinct therapeutic advantage. In such selected patients, complete removal of the affected esophageal segment can not only lead to significant improvement in symptoms such as dysphagia but also holds the potential for a long-term drug-free state, thereby avoiding the side effects and monitoring associated with chronic immunosuppression. Given the advancements in surgical techniques, mediastionoscopy-assisted esophagectomy (MAE) has emerged as a procedure with potentially lower perioperative morbidity and faster recovery compared to traditional open or thoracoscopic surgery. While MAE for esophageal diseases is associated with a higher incidence of recurrent laryngeal nerve palsy compared to thoracoscopic approaches, it confers the significant advantage of reduced pulmonary complications, including pneumonia.^16,17)^ In the present case, the benign nature of the esophageal stricture meant that extensive lymph node dissection was not needed, but early recovery without any complications was a primary concern. Therefore, MAE was selected. Although recurrence after surgery is a possibility if the disease is not truly localized or if resection is incomplete (e.g., strictures post-esophagectomy were reported^5)^), the focused benefit of MAE in reducing pulmonary risk in a COPD patient with localized benign disease made it a compelling option. Nevertheless, while surgical resection may be curative in cases of localized IgG4-related esophageal disease, postoperative follow-up remains essential due to the systemic and potentially relapsing nature of IgG4-RD.^18)^ In the absence of other organ involvement or residual disease, additional immunosuppressive therapy may not be required.^19)^ However, regular clinical monitoring, including periodic imaging and serum IgG4 level assessment, is recommended to detect recurrence or metachronous involvement of other organs. Further studies and accumulation of cases are required to validate the long-term efficacy and safety of MAE for IgG4-related esophageal disease.

## CONCLUSIONS

Diagnosing IgG4-related esophageal stenosis can be difficult. Clinicians should consider it in cases of refractory strictures even with non-diagnostic biopsies, and serum IgG4 testing is advised despite its low sensitivity. In select cases of benign stenosis with diagnostic uncertainty, especially in high-risk patients, mediastinoscopy-assisted esophagectomy can be a useful surgical approach to alleviate symptoms and potentially achieve a drug-free state.

## DECLARATIONS

### Funding

No funding was received for this manuscript.

### Authors’ contributions

MS contributed to the conception and design of this case report, acquisition and interpretation of data, drafted the manuscript, and approved the final version for publication.

MS agrees to be accountable for all aspects of the work.

ST conceptualized and supervised this case report, critically revised the manuscript for important intellectual content, approved the final version for publication, and agrees to be accountable for all aspects of the work.

YT performed the histopathological analysis, provided the pathological images, contributed to the interpretation of the pathological findings, and critically reviewed the manuscript for pathological content.

YT approved the final version for publication and agrees to be accountable for the pathological aspects of the work.

SO, MH, SK, KU, RA, YY, TY, TS, MM, KK, RO, NH, YI, SH, KH, MN, and KO critically revised the article and approved the final version for publication.

All authors have read and approved the manuscript.

### Availability of data and materials

The datasets supporting the conclusions of this article are included within the article.

### Ethics approval and consent to participate

Patient privacy was considered, and the manuscript includes no identifying information. Our institution does not require ethical approval for case reports. Informed consent to participate in this study was obtained from the patient.

### Use of artificial intelligence tools

We did not use any artificial intelligence tools.

### Consent for publication

The patient provided informed consent for the publication of this case.

### Competing interests

All authors declare that they have no competing interests.
